# Aging Increases Hippocampal DUSP2 by a Membrane Cholesterol Loss-Mediated RTK/p38MAPK Activation Mechanism

**DOI:** 10.3389/fneur.2019.00675

**Published:** 2019-06-25

**Authors:** Adrián Martín-Segura, Álvaro Casadomé-Perales, Pietro Fazzari, José Manuel Mas, Laura Artigas, Raquel Valls, Angel R. Nebreda, Carlos G. Dotti

**Affiliations:** ^1^Department of Molecular Neuropathology, Centro de Biología Molecular Severo Ochoa, CSIC/UAM, Madrid, Spain; ^2^Albert Einstein College of Medicine, Bronx, NY, United States; ^3^Centro de Investigación Príncipe Felipe, Valencia, Spain; ^4^Anaxomics Biotech, Barcelona, Spain; ^5^Institute for Research in Biomedicine (IRB Barcelona), The Barcelona Institute of Science and Technology, Barcelona, Spain; ^6^Institució Catalana de Recerca i Estudis Avançats, Barcelona, Spain

**Keywords:** cholesterol, RTKs, p38MAPK, aging, DUSP2

## Abstract

Numerous studies suggest that the increased activity of p38MAPK plays an important role in the abnormal immune and inflammatory response observed in the course of neurodegenerative diseases such as Alzheimer's disease. On the other hand, high levels of p38MAPK are present in the brain during normal aging, suggesting the existence of mechanisms that keep the p38MAPK-regulated pro-inflammatory activity within physiological limits. In this study, we show that high p38MAPK activity in the hippocampus of old mice is in part due to the reduction in membrane cholesterol that constitutively occurs in the aging brain. Mechanistically, membrane cholesterol reduction increases p38MAPK activity through the stimulation of a subset of tyrosine kinase receptors (RTKs). In turn, activated p38MAPK increases the expression and activity of the phosphatase DUSP2, which is known to reduce the activity of different MAPKs, including p38MAPK. These results suggest that the loss of membrane cholesterol that constitutively occurs with age takes part in a negative-feedback loop that keeps p38MAPK activity levels within physiological range. Thus, conditions that increase p38MAPK activity such as cellular stressors or that inhibit DUSP2 will amplify inflammatory activity with its consequent deleterious functional changes.

## Introduction

Brain inflammation is frequently related to several diseases and it has been described to be a conspicuous component of Alzheimer's disease (AD), Parkinson's Disease (PD) and multiple sclerosis (MS) (1) and also of acute situations such as stroke and head trauma (2, 3). In all these conditions the final outcome is usually the loss of neuronal cells. However, different physiological events lead along lifespan to the development of inflammatory processes (4). In this regard, brain inflammation is also evident in the brain during non-pathological aging (5–8), where the loss of neurons is not usual (9, 10). These last observations are consistent with the view that during aging precise mechanisms must be developed to keep the level of activity of the different mediators of the inflammatory process within physiological range.

The inflammation onset is characterized by the increment of inflammatory cytokines (11) together with the activation of several key elements, such as MAPKs (mitogen-activated protein kinases) and other signaling proteins, that allow the progression of the inflammation generating a positive feedback between those two elements. Although there are several signaling proteins that regulate inflammation, one of the main players is the p38MAPK pathway (12). This pathway is a crucial regulator of inflammatory events through several mechanisms including changes in gene expression (12). Furthermore, p38MAPK activation has been related to several neurodegenerative diseases (13, 14). As an important signal integrator pathway, p38MAPK has also been linked to other processes different from inflammation such as development, cell cycle or even memory processes (15). Considering its importance in gene expression modulation, the increased p38MAPK activity observed in physiological brain aging (16) suggests that this pathway could be part of the age-associated mechanisms responsible for maintaining brain inflammation within a physiological range.

In previous studies, we demonstrated that the gradual loss of cholesterol from the neuronal plasma membrane during aging contributes to neuronal survival thanks to the increased activity of pro-survival kinase AKT1 due, among other causes, to the increase in basal activity of tyrosine kinase receptors (RTKs) (17, 18). In addition, our previous works suggested that the constitutive reduction in neuronal plasma membrane cholesterol during aging may be, at least in part, a consequence of increased activation and plasma membrane translocation of the cholesterol catabolic enzyme Cyp46A1 (17, 19). Hence, the recent demonstration that RTK activation favors survival in the developing brain via the p38MAPK pathway (20), moved us to test the hypothesis that reduced membrane cholesterol, via RTKs' stimulation, could contribute to the increase in p38MAPK activation in the old brain.

## Results

### Age Increases p38MAPK Activity Levels in the Hippocampus, in Part Due to Cholesterol Loss

**Figure 1:**
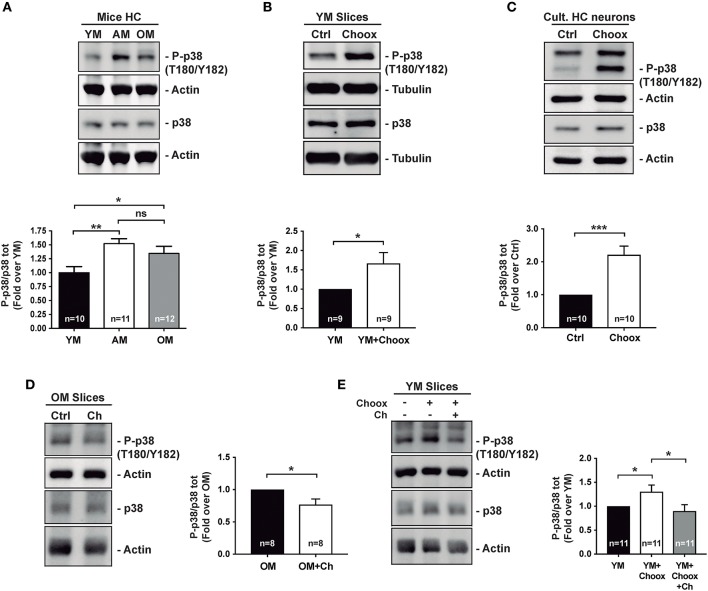
Age increases p38MAPK activity in mice hippocampus in a cholesterol-dependent manner. **(A)** Age-associated increase in the phosphorylation of p38MAPK (p38) activating residues (T180/Y182) in mice hippocampus (HC): young (2–4 months; YM), adult (7–12 months, AM), old (20–24 months OM). **(B)** Increased p38MAPK activating marks (phosphorylation on T180/Y182) in hippocampal slices from 2 month-old mice with reduced cholesterol (treated with cholesterol oxidase, Choox, for 30 min). **(C)** Increased p38MAPK activating marks in hippocampal neurons in culture treated as hippocampal slices in **(B)**. **(D)** Reduced p38MAPK activity in old mice hippocampal slices incubated with a cholesterol replenishment solution (cholesterol-methyl-beta-cyclodextrin complex, referred as Ch). **(E)** Increased p38MAPK activity induced by the cholesterol extracting enzyme Choox become reduced when the same slices are subsequently incubated with the cholesterol (referred as Ch) rich solution. Numbers in bars reflect number of independent experiments. Data are represented as mean ± SEM. The asterisks indicate the *p*-values (*p < 0.05; **p < 0.01; ***p < 0.001. ns, not significant).

Previous work has shown that p38MAPK activity increases with age in the mouse hippocampus (8). Analysis of hippocampal extracts from mice of different ages confirmed that there was a significant increase in active p38MAPK levels between 2–3 and 7–9 months of age, and these remain elevated in 22–24 months-old mice (Figure 1A).

There are also several examples where brain inflammation, in which p38MAPK has a preponderant role, has been associated to the loss of neuronal cholesterol that occurs both in conditions of acute (e.g., stroke) and chronic (aging) inflammation (21, 22). Therefore, we decided to investigate the relationship between p38MAPK increase and neuronal cholesterol loss. As a first approximation, we reduced cholesterol levels in hippocampal slices from young mice by cholesterol oxidase (Choox) treatment (see Materials and Methods). We used Choox at a concentration 10 IU/ml, which based on our previous works is a dose that induces a mild (~20%) reduction of plasma membrane cholesterol, without affecting cell viability (Palomer et al., 2016) (23). Figure 1B shows that a cholesterol decrease of this magnitude increases the levels of the phosphorylated (active) form of p38MAPK in hippocampal slices from young mice. A similar treatment in cultured hippocampal neurons also resulted in a significant increase in p38MAPK activity (Figure 1C), altogether indicating that cholesterol loss can be sufficient for p38MAPK activation.

In order to determine if cholesterol loss is necessary for p38MAPK increase with age (see Figure 1A), we raised the levels of this lipid to hippocampal slices of old mice by adding a solution of cholesterol-methyl-beta-cyclodextrin (MβCD-Ch, referred in figures as Ch). It has been previously shown that the high affinity of methyl-beta-cyclodextrin (MβCD) for cholesterol can be used to generate inclusion complexes that increase membrane cholesterol levels (24, 25). Hippocampal slices from old mice were incubated with MβCD-Ch following protocols used in previous studies in which we evaluated that this treatment restores cholesterol content to levels similar to those of young mice (25, 26). Figure 1D shows that MβCD-Ch significantly reduces the levels of phosphorylated p38MAPK in the old hippocampal slices. Further supporting that cholesterol loss can account for the increased p38MAPK activity in the old slices, the increase due to Choox was restored when the Choox-treated slices from young mice were re-incubated with the MβCD-Ch complex (Figure 1E). Altogether, the results are consistent with the possibility that conditions that lead to a reduction of neuronal cholesterol, acute or chronic, increase p38MAPK activity. The next question we asked was: how does cholesterol loss lead to the activation of p38MAPK?

### RTK Activation Plays a Role in Cholesterol Loss-Mediated p38MAPK Activity Increase

Considering that an acute loss of cholesterol could generate cellular stress, a well-known p38MAPK activator, we checked if the activation of p38MAPK upon cholesterol removal was due to an increase in oxidative stress. To investigate this possibility, hippocampal neurons in culture were incubated with an antioxidant cocktail at the time of the Choox-induced cholesterol reduction (see Materials and Methods). The antioxidant treatment partially prevented the Choox-induced increase in p38MAPK activity (Figure S1A), indicating that still a significant fraction of the p38MAPK activated by cholesterol loss occurs independently from oxidative stress, although this is also induced by cholesterol loss (27).

The loss of cholesterol from the plasma membrane, even if <20% as in our conditions, will necessarily lead to substantial structural changes in the plasma membrane causing a panoply of functional alterations. Therefore, we investigated whether activation of p38MAPK in the neurons with reduced cholesterol was the consequence of a particular type of membrane signaling alteration or, on the contrary, the consequence of multiple altered pathways. In particular, we checked the possibility that different known p38MAPK activators could become active upon cholesterol loss. Incubation of cortical neurons with a cell permeable calcium chelator (BAPTA-AM) did not significantly reduce the p38MAPK increase induced by cholesterol loss (Figure S1B), ruling out Ca^2+^ levels alterations as promoter of p38MAPK activation in this experimental situation. Similarly, incubation of the neurons with H-89, a protein kinase inhibitor with preference for protein kinase A (PKA) (28, 29), or with Chelerythrine-chloride, an inhibitor of PKC isoforms A and B (30), also failed to interfere with the cholesterol loss-induced p38MAPK activation (Figure S1C). These results show that the loss of cholesterol induced by Choox treatment does not have, in itself, such a pleiotropic effect. Given that in previous studies we showed that the loss of cholesterol increases the activity of the RTKs TrkB and insulin receptor (IR) (17, 31), we analyzed next if RTKs were involved in cholesterol loss-mediated p38MAPK activation. As a first approach, we incubated cell lysates of Choox-treated and un-treated cultured hippocampal neurons, with a membrane-based antibody array to determine the relative phosphorylation levels of several mouse RTKs (see Materials and Methods). This study revealed that cholesterol loss was able to increase the phosphorylation levels of different RTKs, most notably SCFR (Stem Cell Factor Receptor) also known and referred here as c-Kit, VEGFR2 (Vascular Endotelial Growth Factor receptor 2), and IGF-1 (Insulin Growth Factor receptor 1) (Figure S2). On the other hand, the phosphorylation levels of several other RTK receptors were not affected by the cholesterol loss (Figure S2), again implying that membrane cholesterol reduction has a limited series of targets, at least at the low levels of reduction induced in our experimental model.

**Figure 2:**
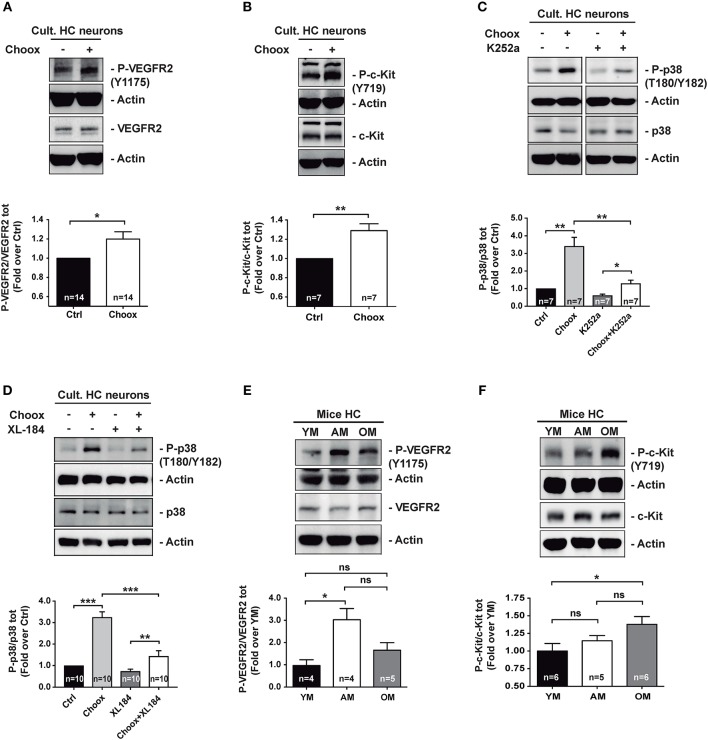
Age-dependent cholesterol loss leads to increased p38MAPK activity through RTKs signaling. **(A)** Increased VEGFR2 activating phosphorylation Y1175 in cultured hippocampal neurons after treatment with cholesterol oxidase (Choox). **(B)** Increased c-Kit receptor activating phosphorylation Y718 in neurons in culture treated with Choox for cholesterol removal. **(C)** Incubation of hippocampal neurons in culture with K252a, a broad RTK inhibitor, significantly prevents Choox-induced p38MAPK (p38) activation. **(D)** Incubation of hippocampal neurons in culture with XL-184, an inhibitor of VEGFR2 and c-Kit receptors, prevents Choox-induced p38MAPK phosphorylation. **(E,F)** Western blots showing activating phosphorylations of RTK receptors, VEGFR2 Y1175 **(E)** and c-Kit Y718 **(F)**, in hippocampus of young (YM), adult (AM), and old mice (OM). The values inside the bars indicate the number of independent experiments. Data are represented as mean ± SEM. The asterisks indicate the *p*-values (ns, not significant; *p < 0.05; **p < 0.01; ***p < 0.001).

In order to validate the antibody array results, we performed western blotting with extracts from cultured hippocampal neurons that had been exposed to the Choox treatment. This experiment confirmed that cholesterol reduction increased VEGFR2 and c-Kit activity (Figures 2A,B). To directly assess the existence of a functional link between cholesterol loss, RTK activation and activation of p38MAPK, we incubated Choox-treated cultured neurons with the broad-spectrum RTK inhibitor K252a (32–34). This treatment significantly prevented p38MAPK activation induced by cholesterol loss (Figure 2C). A similar inhibition of p38MAPK activity was observed using the RTK activity inhibitor Cabozantinib (XL184), a small-molecule kinase inhibitor with potent activity toward c-Kit and VEGFR2 (35, 36), (Figure 2D).

The demonstration of a mechanistic link between cholesterol loss, activation of particular RTKs and activation of p38MAPK in cultured neurons, moved us to ask whether the RTK activity increase was also observed *in vivo*, in the old adult brain, as for p38MAPK activity (see Figure 1). To test this possibility, we analyzed the activity levels of the two RTKs activated by cholesterol loss whose inhibition reduced p38MAPK activation (Figure 2D), namely VEGFR2 and c-Kit. Figures 2E,F show that while the VEGFR2 activity increased significantly from the young age (2–3 months) to adulthood (7–9 months of age), remaining high in the old mice (22–24 months old), the c-Kit increased gradually with age, with the change being most significant between young and old mice. As a whole, these experiments show that the activation of p38MAPK in the hippocampus of old mice could be due to a cholesterol loss-mediated activation of particular RTKs. Hence, we next aimed to identify the downstream targets of p38MAPK.

### Cholesterol Loss-Mediated p38MAPK Activation Increases *Dusp2* Gene Expression

**Figure 3:**
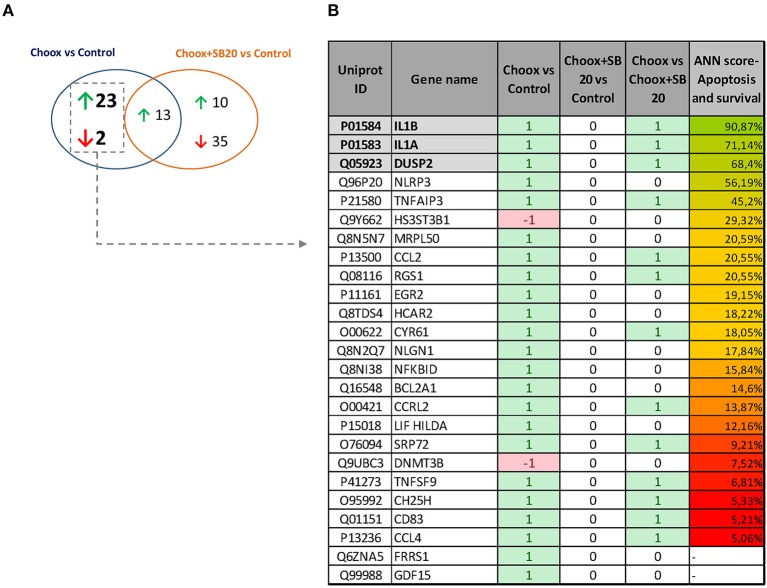
Gene expression changes induced by cholesterol loss in neurons. **(A)** Venn diagram showing the number of genes whose differential expression is statistically significant (*p* < 0.05) upon cholesterol loss (Choox vs. Control) or upon cholesterol loss in the absence of p38MAPK activity (Choox+SB20 vs. Control). Green arrows stand for upregulation (higher expression of the genes in the Choox or Choox +SB20 neurons) and red arrows for downregulation. **(B)** Shows the list of 25 genes whose expression is modified by the loss of cholesterol through a p38MAPK dependent mechanism. It is also indicated if these genes are differentially expressed when statistically comparing the Choox vs. Choox+SB20 cohorts. In the columns referring to the three comparisons, it is indicated if the genes were statistically upregulated (1), downregulated (−1) or if there was no statistically significant change on their expression (0). The column in the right displays the Artificial Neural Network (ANNs) score obtained by each gene when calculating their functional relationship with apoptosis and survival through the analysis of mathematical models.

p38MAPK is a well-known modulator of gene expression (37, 38), and its activation in the old mouse brain could be leading to the expression of different genes involved in aging progression. To determine which genes are regulated by the activity of p38MAPK induced by cholesterol loss, we performed a RNA sequencing (RNAseq) study using cultured hippocampal neurons either untreated or treated with Choox to reduce cholesterol, and incubated with or without the p38MAPK inhibitor SB203580 (Choox+SB20) (39). We reasoned that the expression of the downstream targets of p38MAPK induced by cholesterol loss should be altered by Choox but unchanged in Choox+SB20 conditions. Non-Choox treated neurons were used as controls. The statistical comparison of the mRNA expression levels reported 38 differentially expressed genes, 36 upregulated and 2 downregulated in response to Choox treatment (*q*-value < 0.05) (Figure 3). On the other hand, 58 genes were differentially expressed when comparing Choox+SB20 with control neurons, 23 upregulated and 35 downregulated in Choox+SB20 treated neurons (see Figure 3A). It is not surprising that more genes are affected in this second analysis, as the p38MAPK inhibitor can have effects independently from cholesterol loss-induced p38MAPK activation. Irrespectively, the analysis of these two lists of genes unveiled a set of 25 genes whose expression was modified by the loss of cholesterol through a p38MAPK dependent mechanism, i.e., changing with Choox treatment and the change being suppressed in the Choox+SB20 treatment (Figure 3B).

Next, the above RNAseq data was subjected to a bio-informatic Artificial Neural Network (ANN) score analysis, a strategy that estimates the functional relationship of a gene/protein with a biological process by analyzing mathematical models aimed to simulate the molecular activity of such process (see Materials and Methods). This approach allowed us to identify genes with a potential role in apoptosis and survival. Figure 3B shows that a well-known target of p38MAPK activity, *Interleukin 1* (see Introduction), has the highest predicted value (functional clustering), confirming the accuracy of this bio-informatics tool. In addition, dual specificity protein phosphatase 2 gene (*Dusp2*) also presented high-predicted value. This result was of interest to us as DUSP phosphatases dephosphorylate MAPKs, and therefore have a potential role limiting the extent of the p38MAPK activation that occurs in the old brain (see Figure 1A). Interestingly, the gene expression of another phosphatase of the same family, *Dusp1*, appeared to be altered by cholesterol removal but in a MAPK-independent manner (see Table S1) thus revealing a specific pattern of gene expression in the context of age-related cholesterol loss.

**Figure 4:**
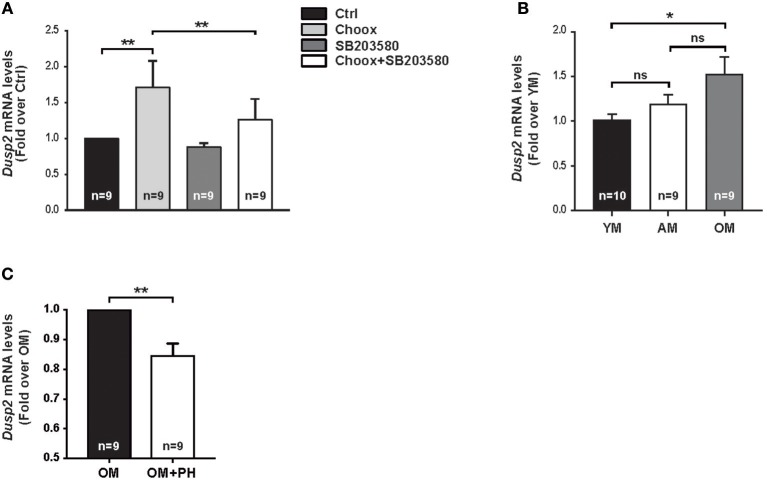
Cholesterol loss-dependent p38MAPK activation increases *Dusp2* mRNA levels in old mouse hippocampus. **(A)** Graphic shows *Dusp2* mRNA levels after cholesterol loss. Hippocampal neurons in culture where treated or not with p38MAPK inhibitor, SB203580 (20 μM), 1 h previous to treatment with Choox for cholesterol removal to address p38MAPK involvement in gene transcription. **(B)** Plot reflects *Dusp2* mRNA levels in the hippocampus of young (YM), adult (AM) and old mice (OM). **(C)** Graphic shows *Dusp2* mRNA levels in hippocampal slices of old mice treated for 1 h with a potent p38MAPK inhibitor, PH787904 (referred as PH; 2 μM). Bar graphs: Numbers inside indicate the number of independent experiments. Data are represented as mean ± SEM. The asterisks indicate de *p*-values (ns, not significant; *p < 0.05; **p < 0.01).

To validate the RNAseq data, we carried out a qPCR study using as template mRNA from 15 days *in vitro* (DIV) hippocampal neurons subjected to Choox treatment in the presence or absence of the p38MAPK inhibitor SB203580. We focused on *Dusp2*, as this gene showed increased levels by cholesterol reduction in a p38MAPK activity-dependent manner and has a high ANN predicted value as survival-apoptosis related genes (see above). These experiments confirmed that cholesterol loss induced the up-regulation of *Dusp2* mRNA in a p38MAPK-dependent manner (Figure 4A).

To investigate if the increase of these mRNAs was the result of the acute loss of cholesterol induced by Choox or can also occur in conditions of gradual cholesterol loss, as during physiological aging, we repeated the qPCR analysis using as template mRNA from the hippocampus of young (2–3 months-old), adult (7–9 months-old) and old (22–24 months-old) mice. Figure 4B shows that *Dusp2* mRNA levels gradually increase with age, reaching significance between young and old mice. This indicates that *Dusp2* upregulation may be the consequence of the gradual changes produced in the plasma membrane during aging. Then, we investigated if the increase with age of *Dusp2* was dependent on the activity of p38MAPK. For this, we incubated hippocampal slices of old mice with the more potent and specific p38MAPK inhibitor PH797804 (40). This experiment resulted in a reduction in the levels of *Dusp2* mRNA (Figure 4C).

**Figure 5:**
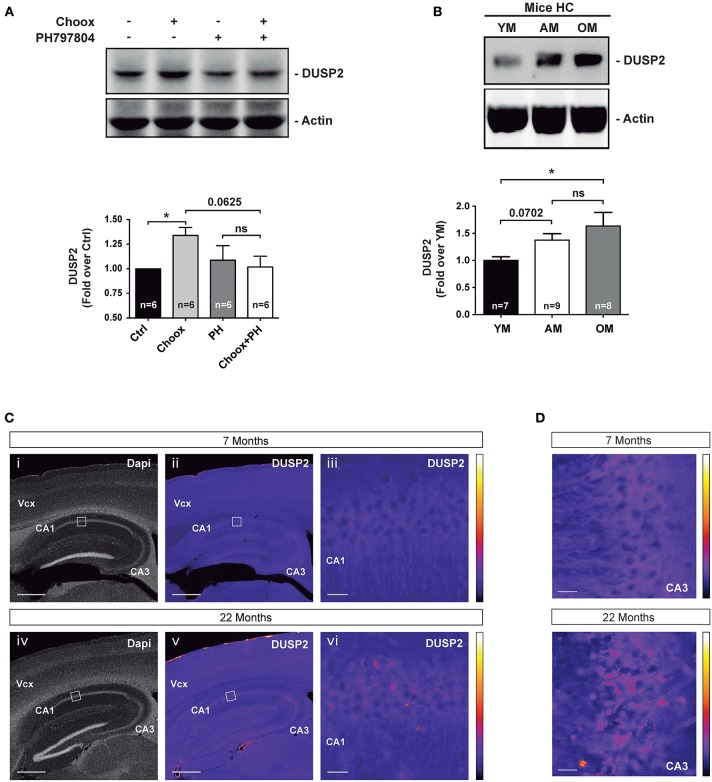
DUSP2 protein levels are upregulated in the hippocampus of old mice. **(A)** DUSP2 levels increase in a cholesterol loss and p38MAPK activity dependent manner. Hippocampal neurons in culture were or were not treated with the p38MAPK inhibitor PH797804 (2 μM, also referred as PH) prior to cholesterol oxidase (Choox). **(B)** DUSP2 protein levels increase with aging in the mouse hippocampus. **(C)** Representative pictures show the increased expression of DUSP2 in the cortex and in the hippocampus with age. Top pictures (i-iii) show expression of DUSP2 in adult mice (7 months); lower pictures (iv-vi) show DUSP2 expression in old mice (22 months). DUSP2 is expressed in CA1 layer (iii; vi). Panels (iii) and (vi) show higher magnifications of the regions boxed in (ii) and (v) respectively. Vcx, Visual cortex; CA1, Cornu Ammonis of the hippocampus layer 1; CA3, Cornu Ammonis layer 3. Scale Bar in i, ii, iv, v, 500 μm; Scale bar in iii, vi, 20 μm. Colored bars on the right show the Look-Up-Table used to color-code the intensity of DUSP2 labeling. **(D)** Pictures show a magnification of CA3 layer. Scale bar represents 20 μm. Bar graphs: Values inside indicate the number of independent experiments. Data are represented as mean ± SEM. The asterisks indicate the *p-*values (ns, not significant; *p < 0.05).

To confirm the increased expression of DUSP2 in the old brain and in cholesterol loss conditions at the protein level, we immunoblotted cell lysates of Choox-treated neurons that had been pre-incubated with the p38MAPK inhibitor PH797804. This experiment confirmed the cholesterol loss and p38MAPK activity-dependent activation of DUSP2 protein (Figure 5A). To test if the increase in DUSP2 observed following cholesterol extraction of cultured neurons was also present *in vivo*, we performed a western blotting analysis using hippocampal extracts from mice of different ages, young, adult and old. In agreement with the mRNA results (see Figure 3B), DUSP2 protein was also found elevated in the hippocampus of old mice compared to young mice (Figure 5B). Immunofluorescence microscopy experiment in hippocampal sections of mice of different ages confirmed this result (Figures 5C,D).

## Discussion

The results presented here have several biological implications. On one hand, they extend previous reports showing that age increases brain p38MAPK activity (8). Mechanistically, our data suggest that during aging the gradual loss of cholesterol leads to the activation of RTKs, which in turn activate p38MAPK signaling. On the other hand, we showed that the cholesterol loss-mediated p38MAPK activation results in the up-regulation of pro-inflammatory genes and also of phosphatases such as *Dusp2* that can potentially limit p38MAPK activity.

DUSP2, originally named phosphatase of activated cells 1 (PAC-1), is one of the members of the dual-specificity phosphatases (DUSPs) that act as negative regulators of MAPKs by dephosphorylating both phosphotyrosine and phosphoserine/threonine residues (41). Since DUSP2 was originally identified in stimulated human peripheral T cells, most of our current knowledge on this phosphatase is on its role in the immune response and inflammation (41, 42). In addition, it has also been proposed that DUSP2 plays a role in apoptosis and cancer (43–45). There are also a few studies on DUSP2 in the central nervous system, for example it has been reported that *Dusp2* mRNA expression is increased in forebrain neurons resistant to ischemia, but not in the vulnerable neurons, suggesting that DUSP2 may be protecting against this type of stress (46). A similar neuroprotective role for DUSP2 has been reported in granule neurons treated with apoptotic stimuli such as cisplatin (47). Thus, the increased expression of DUSP2 in response to cholesterol loss/redistribution, as it occurs in the old brain, could have a dual role in brain physiology: it would maintain p38MAPK activity at physiological levels, so that this pathway can perform its usual functions in synaptic plasticity and cytoskeletal stability, while at the same time it would ensure that the extent of p38MAPK activation is not too exaggerated, which could lead to neuronal damage, such as in stress conditions. The different roles of p38MAPK in physiological vs. pathological situations, might be the consequence of a qualitative process, due to the existence of “pools” of p38MAPK receiving input from different signaling pathways. Alternatively, physiological or pathological responses could be the consequence of a quantitative process and rely on the intensity of the pathway activity induced by different stimuli. Thus, the activity of p38MAPK that leads to the transcription of *Dusp2* could be due to a low intensity stimulus, such as the one elicited by the physiological loss of membrane cholesterol, while in response to stronger stress stimuli the negative regulation by DUSP2 phosphatase could be overcome by higher levels of upstream p38MAPK activators or by other mechanisms leading to enhanced p38MAPK activity and neuronal death. As a matter of fact, although the activity of p38MAPK is significantly higher in the old brain, it is not enough to induce neuronal death nor are there signs of pathological inflammation, suggesting that p38MAPK activity levels may have not reached the disease-producing threshold during normal aging. In addition to the negative regulation of p38MAPK activity, the cholesterol loss-induced upregulation of DUSP2 may also protect old neurons by dephosphorylating ERK1/2, a pathway known to be less active during aging (48, 49). Although a number of studies have shown the ERK1/2 pathway to have an anti-apoptotic role in neurons, pro-apoptosis induced by ERK1/2 signaling has also been observed. Thus, aberrant activation of MEK/ERK signaling induced by β-amyloid peptide promotes the apoptosis of rat embryonic cortical neurons by regulating the entry of neurons into the cell cycle (50). Furthermore, neuronal apoptosis mediated by the Ras/Raf-1/MEK/ERK signaling pathway was also reported in conditions of mitochondrial dysfunction (51) and zinc depletion (52), or when ERK signaling is activated together with JNK (53), when glutamate receptors (NMDA) are activated by tumor necrosis factor (54), and in conditions of sustained ERK activity (55).

In conclusion, we propose that cholesterol loss-mediated DUSP2 expression during physiological aging may be part of a protective signaling mechanism to regulate p38MAPK over-activation in neurons. In this hypothetical scenario, impaired expression of DUSP2 could facilitate the exacerbation of p38MAPK-mediated responses, thus contributing to the development of pathologies like AD, PD or MS. Future studies will be required to explore this working hypothesis.

## Materials and Methods

### Animal Handling

Male C57BL/6J mice were used in this study: young 2–3 month-old, adult 7–12 month-old and old 20–24 month-old. All the animals were kept in the Centro de Biología Molecular Severo Ochoa's (CBMSO) animal facility. The mice and manipulations presented in this work count with the approval of the Dirección General de Medio Ambiente de la Comunidad Autónoma de Madrid (Ref. PROEX 066/15) and the CBMSO's Ethical Committee. All the experiments were performed in accordance with European Union guidelines (2010/63/UE) regarding the use of laboratory animals.

### Cell Cultures

Primary hippocampal neurons were extracted from Wistar rat embryos at embryonic day 18 (E18), seeded in culture conditions as previously described (56) and kept in culture for 15 days *in vitro* (DIV). All cells were incubated at 37°C, humidity conditions and 5% CO_2_.

### Hippocampal Slices

Hippocampal slices were obtained from C57BL/6J mice. Hippocampi were extracted and placed in dissection solution (10 mM D-glucose, 4 mM KCl, 26 mM NaHCO_3_, 233.7 mM sucrose, 5 mM MgCl_2_, 1:1000 Phenol red) oxygen saturated with carbogen (95% O_2_/5% CO_2_), and sliced using an automatic tissue chopper (McIlwain Tissue Chopper, Standard Table, 220 V, Ted Pella Inc.) to obtain 400 μm hippocampal slices. Then slices were kept in artificial cerebrospinal fluid (ACSF: 119 mM NaCl, 2.5 mM KCl, 1 mM NaH_2_PO_4_, 11 mM glucose, 1.2 mM MgCl_2_, 2.5 mM CaCl_2_, osmolarity adjusted to 290 Osm) oxygen saturated with carbogen for 1 hour. Experiments were performed in ACSF oxygen saturated.

### Cell and Slices Treatments

The following compounds were added to cell medium of hippocampal neurons: Cholesterol oxidase (Choox; Calbiochem ref.: 228250; 10 IU/ml); K252a (Tocris ref.: 1683; 1 μM); SB203580 (Shelleckchem ref.: S1076; 20 μM); PH797804 (Axon Medchem ref.: 1837; 2 μM); H89 (Tocris ref.: 2910; 50 μM); Chelerythrine (Tocris ref.: 1330; 10 μM); XL-184 (Tocris ref.: 5422; 1 μM); BAPTA-AM (Invitrogen ref.: B-6769, 10 μM). Experiments with the antioxidants cocktail in cultured hippocampal neurons used: N-Acetyl-L-Cysteine (NAC, Sigma-Aldrich ref.: A7250, 5 mM) and L-Glutathione reduced (GSH, Sigma-Aldrich ref.: G4251, 5 mM). Hippocampal slices were treated with Cholesterol oxidase (Choox; Calbiochem ref.: 228250; 10 IU/ml) for cholesterol removal. Experiments for cholesterol addition conducted in hippocampal slices were performed at 25°C. Methyl-β-cyclodextrin-cholesterol (MβCD-Ch) solution was prepared freshly at use concentration in ACSF, containing 30 μM Cholesterol Water-soluble (Sigma-Aldrich ref.: C4951) and 5 μM Cholesterol (Sigma-Aldrich ref.: C3045).

### Antibodies

The following antibodies were used for western blot (WB) and immunofluorescence (IF): anti-α-Tubulin (WB 1:10000, Abcam ref.: ab7291), anti-β-Actin (WB 1:20000, Sigma-Aldrich ref.: A5441), anti-Phospho p38MAPK (T180/Y182) (WB 1:1000, Cell Signaling ref.: #4511), anti-p38MAPK (WB 1:1000, Abcam ref.: ab170099), anti-Phospho VEGFR2 (Y1175) (WB 1:1000, Cell Signaling ref.: #2478), anti-VEGFR2 (WB 1:1000, Cell Signaling ref.: #3770), anti-Phospho c-Kit (Y719) (WB 1:1000, Cell Signaling ref.: #3391), anti-c-Kit (WB 1:1000, Cell Signaling ref.: #3074 and WB 1:1000, Santa Cruz ref.: sc-13508), anti-DUSP2 (WB 1:1000, IF 1:100, Sigma-Aldrich ref.: SAB4300841), PathScan® RTK Signaling Antibody Array Kit (Chemiluminescent Readout, Cell Signaling ref.: #7982).

### Relative RT-PCR

Cultured hippocampal neurons or mice hippocampi were homogenized in Trizol Reagent (Life Technologies ref.: 15596018) and the RNA was extracted using Direct-zolTM RNA minipreps (Zimo research ref.: R2052). RNA was quantified at 260 nm absorbance using a Nanodrop ND-100 (Themo Fisher Scientific). First strand cDNA was obtained using RevertAid H Minus First Strand cDNA Synthesis kit (Themo Fisher Scientific ref.: K1631). 5 ng of synthesized cDNA were used to perform the qPCR using GoTaq® qPCR Master Mix (Promega ref.: A6002) in ABI PRISM 7900HT SDS (Applied Biosystems; Life Technologies). Primers obtained from Sigma-Aldrich were used at 0.5 μM final concentration (see list below). Three housekeeping genes *Gapdh, Gus-B* and *Pgk-1* were used as endogenous controls.

### Primers

Rat primers used for qPCR in neurons in culture:

*Gapdh* forward: 5′- ATGACTCTACCCACGGCAAG -3′

*Gapdh* reverse: 5′- GATCTCGCTCCTGGAAGATG -3′

*Gus-B* forward: 5′- GCCAATGAGCCTGTCTCTTC -3

*Gus-B* reverse: 5′- TCCAGTTCTTGGGGAATCTG -3′

*Pgk-1* forward: 5′- AATGATGCTTTTGGGACTGC -3′

*Pgk-1* reverse: 5′- TCAAAAATCCACCAGCCTTC -3′

*Dusp2* forward: 5′- CCCGAGGGTTCCTATCTATG -3′

*Dusp2* reverse: 5′- AGGGCAAGATTTCCACAGG -3′

Mouse primers used for qPCR in hippocampal samples:

*Gapdh* forward: 5′- CTCCCACTCTTCCACCTTCG -3′

*Gapdh* reverse: 5′- CATACCAGGAAATGAGCTTGACAA -3′

*Gus-B* forward: 5′- AGCCGCTACGGGCGTCG -3′

*Gus-B* reverse: 5′- GCTGCTTCTTGGGTGATGTCA -3′

*Pgk-1* forward: 5′- TACCTGCTGGCTGGATGG -3′

*Pgk-1* reverse: 5′- CACAGCCTCGGCATATTTCT -3′

*Dusp2* forward: 5′- CCGAGGGTTCCGATCTATGA -3′

*Dusp2* reverse: 5′- TAGGGCAAGATTTCCACAGG -3′

### mRNA Sequencing Data

Hippocampal neurons where treated or not 30 min with Cholesterol oxidase (10 IU/ml) after 1 h treatment either with DMSO or the broad p38MAPK inhibitor SB203580 (20 μM). Total RNA was extracted as described for Relative RT-PCR. A differential gene expression analysis of the RNA extracted was performed by GATC Biotech (InView Transcriptome Advance; GATC Biotech) on a Genome Sequencer Illumina HiSeq2500 (HiSeq Rapid Run, 50 bp paired end). Gene expression was analyzed using the Bowtie, TopHat, Cufflinks, Cuffmerge, Cuffdiff software suite.

For the subsequent biocomputational analyses, the differentially expressed rat genes were converted into the corresponding human equivalent UniProt reviewed protein according to the following steps: (i) UniProt ID automatic crossing of the rat proteins with human proteome with corresponding databases (57), (ii) gene name automatic crossing of the rat genes with human genes (Gene Name in UniprotKB database) and (iii) Manual Blast (58), selecting the best reviewed match presenting at least an identity value ≥70% and E-value 10^−6^.

### Artificial Neural Network (ANN) Score Analysis

The possible molecular relationship between the differentially expressed genes and apoptosis and survival was evaluated by means of artificial neuronal networks (ANNs), following TPMS technology protocols (59, 60). This approach involves the generation of mathematical models of the biological processes through the use of artificial intelligence techniques, a methodology involving three steps: (i) the molecular characterization of apoptosis and survival according to bibliography to identify key effector proteins currently associated with these processes (databases: PubMed, ScienceDirect and Scopus), (ii) the generation of a protein-protein map (physical interactions or functional relationships) around these key effectors using information stored in public databases (e.g., Reactome, MINT, BioGrid) and (iii) the transformation of the protein map into mathematical models by training it with a collection of known input-output physiological signals was used obtained from literature mining and a compendium of databases that accumulates biological and clinical data (61).

Then, mathematical models of apoptosis and survival were solved by ANNs, which are supervised algorithms that identify relationships between the different nodes in the network. ANN analysis yields a score for each differential gene based on the validations of the prediction capacity of the mathematical models toward known drugs and diseases, as described in databases. The higher the score, the stronger is the predicted mechanistic relationship between the evaluated protein and the biological process. Each score is associated with a *p*-value that describes the probability of the result being a true positive one.

### Immunofluorescences

Immunofluorescences were performed according to standard procedure. Briefly, brains were perfused with PBS and postfixed in 4%PFA-PBS, cryoprotected and cut sagittal at 40 μm at the cryostat. Sections were incubated with rabbit anti-DUSP2 antibody (SAB4300841, Sigma-Aldrich) diluted 1/100 in 2%BSA-0.1%TritonX100-PBS at 4 degrees for 48 h; next, with a donkey anti-rabbit antibody conjugated with Alexa555 (ThermoFisher, A-31572) diluted 1/500 and DAPI 1/2000 (Merck, 268298) in 2%BSA-0.1%TritonX100-PBS. Pictures were taken in identical conditions for the various samples on a microscope Zeiss Cell Observer, with a camera ORCA-Flash4.0 LT sCMOS (C11440-42U) (Hamamatsu). For low magnification we used a 5X/0.15 Plan-Neofluar dry; for high magnification, 25X/0.8 Plan-Neofluar Oil. Images were processed with ImageJ software to adjust luminosity with identical parameters for control and experimental conditions. The look-up-table “Fire” of ImageJ was used for color coding.

### Statistical Analyses

Statistical analyses were performed with Graphpad Prism 5 (Graphpad Software Inc.). All values of the independent experiments are presented as mean ± S.E.M. (standard error of the mean). The numbers of biological replicates are indicated in each figure. Data normality and variances were tested by Shapiro-Wilk test. Student's *t*-test was used for statistical analysis of parametric data. Mann-Whitney *U*-test was used for non-parametric data. Asterisks in the figures indicate *p*-values as follows: ^*^ <0.05; ^**^ <0.01; ^***^ <0.001.

## Data Availability

All datasets generated for this study are included in the manuscript and the Supplementary Files.

## Ethics Statement

The mice and manipulations presented in this work count with the approval of the Dirección General de Medio Ambiente de la Comunidad Autónoma de Madrid (Ref. PROEX 066/15) and the CBMSO's Ethical Committee: Dr. José Fernández Piqueras, Dr. José Javier Lucas, Dra. Elena Hevia, Dra. Carmen Fernández and D. Fernándo Nûñez. All the experiments were performed in accordance with European Union guidelines (2010/63/UE) regarding the use of laboratory animals.

## Author Contributions

AM-S and CD contributed to the design of the different experiments. AM-S, ÁC-P, and PF performed the experimental work. JM, LA, and RV performed the RNA seq analysis. AM-S did the statistical analysis. AN contributed with experimental suggestions and data interpretation. CD, AM-S, and AN prepared the manuscript. CD is the guarantor of this study.

### Conflict of Interest Statement

The authors declare that the research was conducted in the absence of any commercial or financial relationships that could be construed as a potential conflict of interest.

## Supplementary Material

Figure S1 Regulation of cholesterol loss-dependent p38MAPK activation in hippocampal neurons. **(A)** Western blots showing p38MAPK (p38) activation (phosphorylation of residues T180/Y182) in hippocampal neurons in culture after cholesterol removal in presence or absence of antioxidants (referred as Antiox.; N-Acetyl-L-Cysteine 5 mM and Glutathione reduced 5 mM) incubated 15 min previous to Choox treatment. **(B)** Western blots of hippocampal neurons in culture show p38MAPK phosphorylation in residues T180/Y182 upon cholesterol depletion in presence or absence of calcium chelator BAPTA-AM (10 μM) 1 h incubation before Choox treatment. **(C)** Blot analysis, in cultured hippocampal neurons, of p38MAPK activating marks (phosphorylated T180/Y182) using PKC (H89 50 μM, left images) or PKA (Chelerythrine 10 μM, referred as Chelery., right images) inhibitors 1 h previous to cholesterol removal treatment. Numbers in bars reflect number of independent experiments. Data are represented as mean ± SEM. The asterisks indicate the *p*-values (^*^*p* < 0.05; ^**^*p* < 0.01; ns, not significant).

Figure S2 Identification of RTKs activated by cholesterol loss in hippocampal neurons. Detail of the RTKs protein array (Cells signaling ref.: #7982) top part left, showing an example of RTKs whose activity state is being modified by cholesterol loss (after incubation with Choox) in hippocampal neurons in culture. Magnification of some representative examples are shown on the top-right part of the figure. The graphics at the bottom of the figure show how the activity state of the representative RTKs change upon cholesterol depletion in hippocampal neurons in culture.

Table S1 The list of the genes differentially expressed in the comparisons Ctrl vs. Choox, Ctrl vs. Choox+SB203580 and Choox vs. Choox+SB203580, according to the analysis of the RNA sequencing experiment in hippocampal neurons in culture. Gene ID, fold change, *p*-value and *q*-value of the comparisons are included in the tables. The RNA sequencing experiment was performed in cells treated for 30 min with Choox for cholesterol removal, as previously described. Cells were treated or not 1 h before with a known p38MAPK inhibitor (SB203580, 20 μM) in order to determine the effect of p38MAPK cholesterol loss-dependent upregulated activity on the changes observed in genes expression.
