# Supplementary figures and images for: Aging Increases Hippocampal DUSP2 by a Membrane Cholesterol Loss-Mediated RTK/p38MAPK Activation Mechanism
Source: Front Neurol. 2019 Jun 25;10:675. doi: 10.3389/fneur.2019.00675 (PMC6603139; doi:10.3389/fneur.2019.00675)

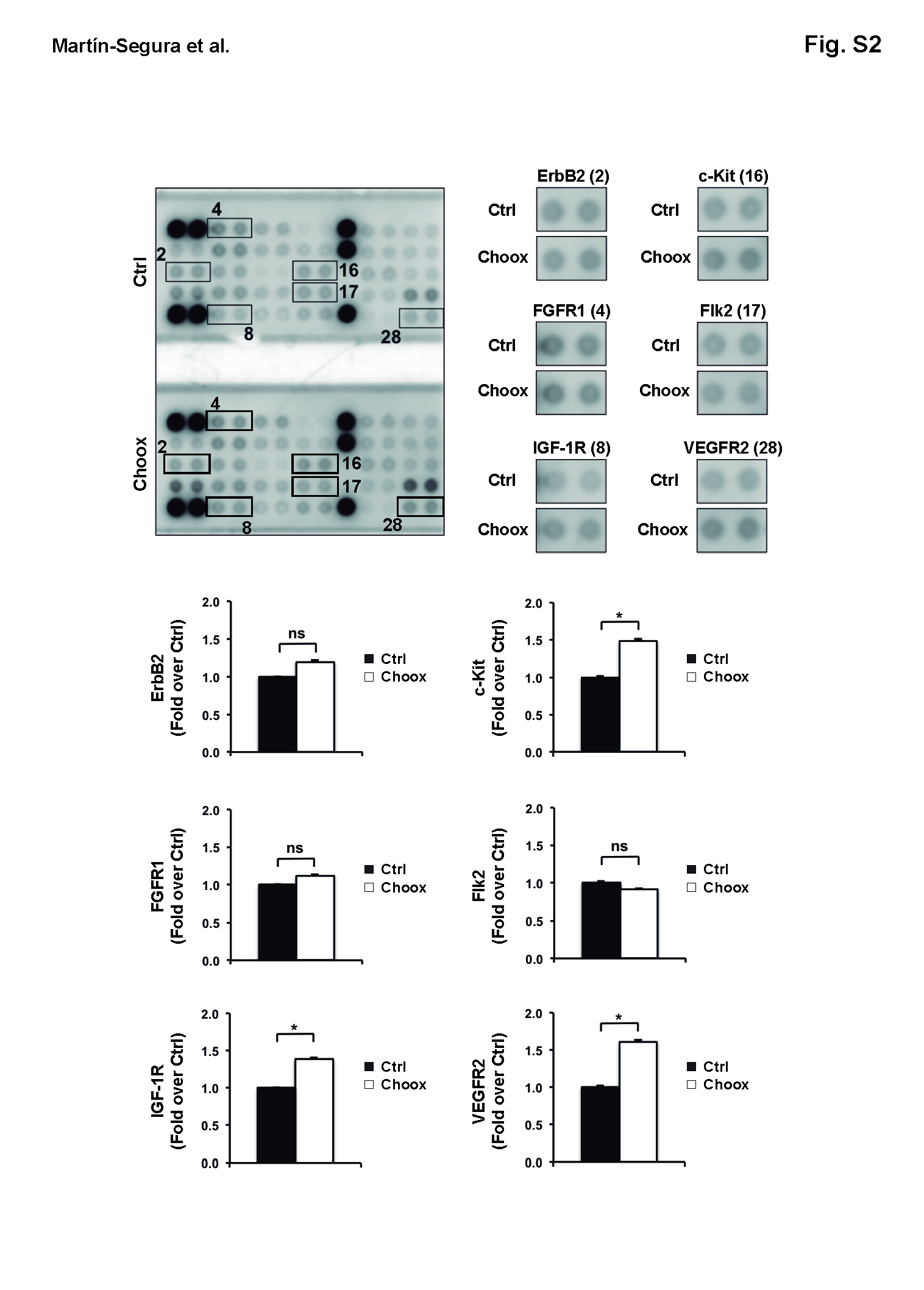

Supplement: Figure S2 — Identification of RTKs activated by cholesterol loss in hippocampal neurons. Detail of the RTKs protein array (Cells signaling ref.: #7982) top part left, showing an example of RTKs whose activity state is being modified by cholesterol loss (after incubation with Choox) in hippocampal neurons in culture. Magnification of some representative examples are shown on the top-right part of the figure. The graphics at the bottom of the figure show how the activity state of the representative RTKs change upon cholesterol depletion in hippocampal neurons in culture. [file Image_2.TIFF]
